# ENT audit and research in the era of trainee collaboratives

**DOI:** 10.1007/s00405-018-5009-1

**Published:** 2018-05-26

**Authors:** Matthew E. Smith, John Hardman, Matthew Ellis, Richard J. Williams, INTEGRATE, The UK National ENT Trainee Research Network

**Affiliations:** 1 Clinical Research Associate and Specialty Registrar in ENT, Addenbrooke’s Hospital, Cambridge, UK; 2 Specialty Registrar in ENT, Royal Marsden Hospital, London, UK; 3 Specialty Registrar in ENT, Freeman Hospital, Newcastle, UK; 4 Specialty Registrar in ENT, Institute of Naval Medicine, Gosport, UK

**Keywords:** Research, Audit, Clinical trial, Surgical training

## Abstract

Large surgical audits and research projects are complex and costly to deliver, but increasingly surgical trainees are delivering these projects within formal collaboratives and research networks. Surgical trainee collaboratives are now recognised as a valuable part of the research infrastructure, with many perceived benefits for both the trainees and the wider surgical speciality. In this article, we describe the activity of ENT trainee research collaboratives within the UK, and summarise how INTEGRATE, the UK National ENT Trainee Research Network, successfully delivered a national audit of epistaxis management. The prospective audit collected high-quality data from 1826 individuals, representing 94% of all cases that met the inclusion criteria at the 113 participating sites over the 30-day audit period. It is hoped that the audit has provided a template for subsequent high-quality and cost-effective national studies, and we discuss the future possibilities for ENT trainee research collaboratives.

The need for evidence-based medicine has been understood for some time [1], though developing the evidence for existing and new interventions can be problematic. Surgical research in particular is often complex and costly to deliver, a problem often exacerbated if multiple institutions are involved. Furthermore, research often requires multidisciplinary input from other health professionals, statisticians, epidemiologists, health economists and patients, and these teams can be difficult to establish.

Surgical trainees face significant hurdles when attempting to participate in, or generate, high-quality research or audit projects. Despite significant enthusiasm and effort, projects may struggle to collect data, remain unfinished or unpublished, or they may simply duplicate work done elsewhere. Individual trainee or small group endeavour is insufficient for the delivery of large high-quality prospective studies, particularly in the context of frequent trainee rotation between institutions and competing clinical demands. The difficulty associated with obtaining research funding and ethical governance approvals further complicates the process. Despite these issues, trainees are required to demonstrate understanding and involvement in audit and research to obtain the certificate of completion of training (CCT). Consultant appointment panels are also advised to look for engagement in clinical research, including trial protocol design and recruitment, rather than simply focusing on first-author publications [3].

## Trainee collaboratives

The delivery of research and audit projects by trainee research collaboratives offers a potential solution to many of the previously mentioned barriers to success. These groups have proliferated in recent years, in particular within the UK, taking advantage of the strength that comes from a network of enthusiastic individuals distributed across the country to deliver audit and research projects on a large scale [2]. Specialty-specific national trainee collaboratives are now common, and EuroSurg has even created a Europe-wide network of medical students and surgeons.

In recognition of their potential, trainee collaboratives and networks have been identified by the Royal College of Surgeons of England as a key part of the research infrastructure being developed to expand the UK surgical clinical trials portfolio [3].

Surgical trainees are ideally suited to participate in clinical research during their training. Their rotational attachments provide an effective multicentre network with significant insight into variations in management, and frontline placement allows interaction with emergency admissions often much sooner than their consultant colleagues. Trainees are also likely to encounter patients at all stages of their surgical journey, from clinic, to consent, to post-operative management and follow-up. For these reasons, a number of trainee collaborative projects have centred on emergent pathology, where this approach can have the edge over conventional trial designs.

Participation in collaborative research has been recognised as a valuable part of surgical training [4], with further incentive for the trainee in the form of high-quality publications, as the collaborative model usually involves publication under a single organisational author, with all participating trainees cited as co-authors.

## ENT trainee collaboration: how we do it

At the start of 2017 ENT training deaneries were surveyed, with data collected from 16 of the 17 regions. Half of these regions (8/16) were found to have a formal research collaborative in place, while around a third (5/16) had ongoing informal trainee collaborations and roughly a fifth (3/16) had no collaboration. Across the collaboratives, eight cohort or observational research projects and 24 audits were underway or completed. Notable successes have included three multicentre audits; retrospective tonsillectomy [5] and pinna haematoma [6] audits, and the prospective quinsy management audit which was the first within the specialty to establish the feasibility of large-scale online data collection [7]. An ENT trainee collaborative has yet to deliver a randomised controlled trial, though they are in development.

INTEGRATE, the National ENT Trainee Research Network, was established in 2015 to deliver national collaborative research projects and audits, and to link together the existing regional ENT trainee collaboratives. The National Audit of Epistaxis Management was the first trainee-led UK-wide collaborative project to be delivered by INTEGRATE, serving as a model for future projects. Epistaxis is one of the most commonly managed conditions within our speciality but evidence-based management guidelines were lacking.

The first stage involved standard setting, with a comprehensive review of the literature surrounding epistaxis facilitated by a network of trainees. Seven research domains related to the management of epistaxis were defined, comprising 15 individual systematic reviews. Eighteen trainees completed the reviews using a common methodology developed by two librarian search specialists and the INTEGRATE team. In total, 49,521 articles were individually screened, with 188 articles included for data extraction.

To generate consensus recommendations on epistaxis management, a dedicated meeting was arranged. Evidence from each research domain was presented to a multidisciplinary panel incorporating patient representatives and utilising the AGREE II framework [8]. These recommendations are currently being assessed in the context of inferential statistical analysis of audit data to generate evidence-based management guidelines for the in-hospital treatment of epistaxis.

The INTEGRATE website (https://www.entintegrate.org) was central to delivery of the audit. Step-by-step guides, standardised information sheets, project protocols and template project registration documents were distributed via the website in addition to advertising material. A custom online portal was used to manage the process of prospective collaborators submitting a site lead application, agreeing to the requirements of the role and obtaining steering committee approval. This portal also formed the basis of mass communication between site leads, regional representatives and the steering committee. Site leads were required to use an online task list to sign off key stages in the process of setting up the project at their centre, allowing the steering committee to identify any issues at an early stage. A structure of regional project champions working in close contact with regionally assigned steering committee members provided an efficient means of management, combining regional knowledge and centralised oversight.

During the data collection period of the audit, online data collection was made possible through the use of a REDCap [9] server (https://www.project-redcap.org) hosted in a secure data safe haven which is compliant with ISO27001 security standards. Automatic data validation was performed prior to submission of each case to ensure complete high-quality data were collected. Only anonymised data were submitted and handled by the steering committee. A study key was held by collaborators at each site in centres to allow appropriate follow-up data to be collected, and these keys were stored and disposed of in line with local clinical governance procedures at each site.

During the prospective audit, 1826 cases were uploaded to the database, representing 94% of all cases that met the inclusion criteria at the 113 participating sites over the 30-day audit period. The submitted data were of high quality, with complete 30-day follow-up data submitted for most cases. Funding from ENT UK allowed commissioned statistical analysis of the data. All participating trainees gained authorship on all papers resulting from the data, as has become the standard practice with trainee collaborative research.

One of the most striking achievements of the epistaxis audit was the low cost of delivery, arising from the streamlined online data collection and management. Total expenditure for the audit and associated consensus process was less than £6000, with previously delivered national audits in the specialty costing more than 10 times this amount [10, 11].

The evidence appraisal, consensus process and audit have provided a tried-and-tested and repeatable model for gaining a greater understanding of how we should manage some of the most common conditions in ENT where considerable variation currently exists. Lessons have been learnt during the audit, in particular relating to how data flow should be managed to ensure patient confidentiality is maintained while facilitating data entry and preventing case duplication. The project management software developed for the epistaxis audit now forms the basis for administration of other regional and national projects, has been made available to collaboratives in other specialties, and has recently been developed to facilitate robust patient anonymisation for future projects.

## The future of INTEGRATE and trainee collaboratives for ENT research and audit

It is hoped that regional and national collaboratives such as INTEGRATE will bring significant benefits to the speciality, directing trainees towards worthwhile projects that align with the key research priorities for the speciality and away from low-impact publications that do little educate the authors nor expand the evidence base. Smaller surgical specialities, including ENT, particularly benefit from national coordination of trainee research. INTEGRATE aims not only to develop and run its own audit and research projects but also to support a portfolio of studies run by individuals or regional collaboratives. For these portfolio studies, INTEGRATE can provide access to a UK-wide network of trainees, experienced consultants, academics, statisticians and methodologists who have volunteered their support and peer review. The network is open to international collaboration, and other collaboratives have led the way in this respect [12]. Additionally, INTEGRATE has organised critical appraisal and research methodology training to prepare trainees for future project involvement.

The national epistaxis audit has generated new research questions and the future for INTEGRATE will include further high-quality prospective observational research, and ultimately randomised controlled trials. Trainees in other specialties have already demonstrated that by working with experienced academics and clinical trials units, trainee collaboratives can gain access to the required expertise and infrastructure to deliver multicentre randomised controlled trials. ROSSINI was the first trainee collaborative-led randomised controlled whereby the West Midlands Surgical Trainee Collaborative successfully enrolled 760 patients into a trial of wound edge protection devices and completed ahead of schedule [13].

Funding is a hurdle for all research. It is a particular issue for trainees who may require relatively modest amounts but who do not have the time or experience to complete application processes. We have demonstrated with the epistaxis audit that trainee collaboration also has the potential to deliver UK-wide audits at very low cost. For trials adopted into the INTEGRATE portfolio, we aim to circumvent the need for funding in some cases through the provision of centralised online data management and by linking together collaborators able to provide statistical or IT support. For larger studies, there is a clear appetite from funding bodies to support trainee collaborative-led research, with the neurosurgical Rescue ASDH trial awarded £1.4 m by the National Institute for health research (NIHR).

The enthusiasm of ENT trainees to participate in collaborative research has been clearly demonstrated by the epistaxis audit and evidence appraisal. Trainees involved in the research network will become consultants who are well equipped to understand, participate in or lead research, and involvement in national or portfolio trials may bring trial money and expertise to centres that have previously not engaged with experimental research. In this way, we hope that INTEGRATE and regional collaboratives will strengthen the culture of research within ENT.
